# Challenges of Periodontal Tissue Engineering: Increasing Biomimicry through 3D Printing and Controlled Dynamic Environment

**DOI:** 10.3390/nano12213878

**Published:** 2022-11-02

**Authors:** Ilaria Roato, Beatrice Masante, Giovanni Putame, Diana Massai, Federico Mussano

**Affiliations:** 1 Bone and Dental Bioengineering Laboratory, CIR-Dental School, Department of Surgical Sciences, University of Turin, 10126 Turin, Italy; 2 PolitoBIOMed Lab and Department of Mechanical and Aerospace Engineering, Politecnico di Torino, 10129 Turin, Italy; 3 Interuniversity Center for the Promotion of the 3Rs Principles in Teaching and Research, 10129 Turin, Italy

**Keywords:** periodontitis, mesenchymal stem cell, mechanical loading, periodontal ligament regeneration, tissue engineering

## Abstract

In recent years, tissue engineering studies have proposed several approaches to regenerate periodontium based on the use of three-dimensional (3D) tissue scaffolds alone or in association with periodontal ligament stem cells (PDLSCs). The rapid evolution of bioprinting has sped up classic regenerative medicine, making the fabrication of multilayered scaffolds—which are essential in targeting the periodontal ligament (PDL)—conceivable. Physiological mechanical loading is fundamental to generate this complex anatomical structure ex vivo. Indeed, loading induces the correct orientation of the fibers forming the PDL and maintains tissue homeostasis, whereas overloading or a failure to adapt to mechanical load can be at least in part responsible for a wrong tissue regeneration using PDLSCs. This review provides a brief overview of the most recent achievements in periodontal tissue engineering, with a particular focus on the use of PDLSCs, which are the best choice for regenerating PDL as well as alveolar bone and cementum. Different scaffolds associated with various manufacturing methods and data derived from the application of different mechanical loading protocols have been analyzed, demonstrating that periodontal tissue engineering represents a proof of concept with high potential for innovative therapies in the near future.

## 1. Introduction

The periodontium is a complex system composed of gingiva, periodontal ligament (PDL), cementum, and alveolar bone, featuring a hierarchically compartmentalized architecture [1]. The homeostasis of this system is maintained by the PDL, a specialized connective tissue, which is located between the cementum and alveolar bone and articulates (gomphosis) the teeth to the jaws [2,3]. Embryologically, PDL derives from the dental follicle cells under the guidance of Hertwig’s epithelial root sheath (HERS), which secrete numerous epithelium-derived factors [4] before obliterating almost completely.

**Figure 1:**
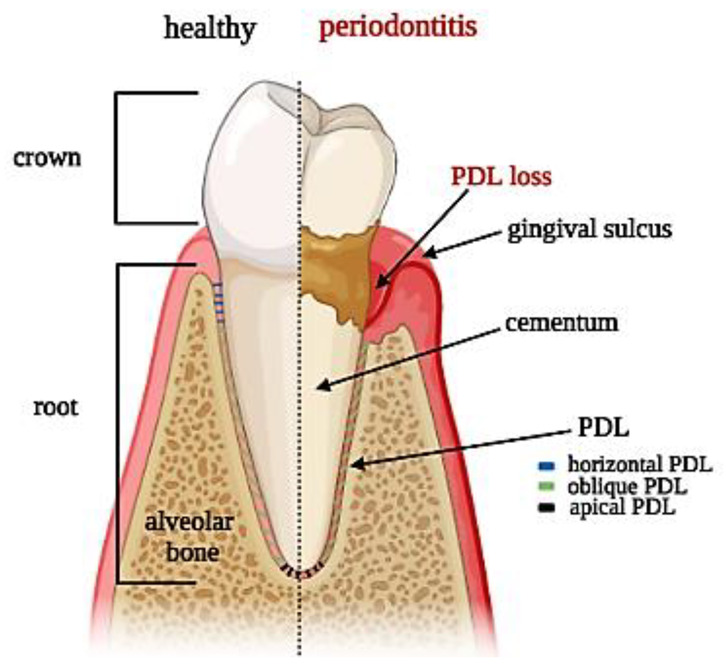
Tooth section showing periodontal ligament anatomy: superficial and deep periodontium along with the typical distribution of PDL fibers in both physiological and pathological conditions.

From a histological perspective, PDL is an aligned fibrous network with a thickness ranging between 100 and 400 µm and is characterized by an extensive blood supply and a neural network [5]. PDL is constituted by a heterogeneous population of cells (namely PDL cells) that includes periodontal ligament fibroblasts (PDLFs), which represent by far the largest population and are responsible for the deposition and maintenance of the extracellular matrix (ECM) and periodontal ligament stem cells (PDLSCs), showing both osteogenic and tendo/ligamentogenic characteristics. Collagen type I and, in lesser amounts, type III constitute cross-banded fibrils, named Sharpey’s fibers, that provide mechanical support and are usually classified as dentinogingival, transseptal, or alveolodental (forming the bulk of proper PDL fibers) [5]. In particular, fibers oblique or perpendicular to the long axis of the tooth are thought to play pivotal roles in eliciting adaptive responses during mastication and occlusion [5]. Among all the fibers, the horizontal ones withstand the greatest loads and exhibit the greatest strain under mastication [6], (Figure 1). The collagen fibers are generally aligned according to a periodic crimped pattern [7] that prevents ligament overextension [8,9]. Sharpey’s fibers anchor mostly to acellular cementum, a mineralized layer (50–300 μm thickness) covering the tooth dentin surface. PDL cells are arranged along PDL fibers so that the long cellular axis is parallel to the main fiber bundles of the PDL [10,11]. The presence of a particular type of elastic fibers named oxtytalan, made of fimbrillins, that form a network running parallel to cementum and are thought to interact with vessels and neural fibers is also noteworthy [12]. In a healthy subject, PDL covers the tooth root almost entirely, and a tight epithelial seal within the gingival sulcus prevents microorganisms from reaching the PDL. This delicate system is compromised by the onset of periodontal disease (PD), which affects in its severe form about 10% of adults, ranking sixth among the most prevalent diseases in the world [13]. PD starts as a localized and reversible inflammation of the gingiva (gingivitis) due to dental plaque, and, when untreated, it may become chronic periodontitis, which is characterized by the progressive destruction of the tooth-supporting tissues, i.e., cementum, PDL, and bone [14,15].

The complex architecture of the periodontal apparatus (Figure 1), including dual-tissue interfaces (alveolar bone–PDL and PDL–cementum of the tooth root), is difficult to regenerate due to the small dimensions of the PDL and the challenging oral environment [16]. Any strategy aiming at periodontal regeneration should entail studying the specific events that guide the formation and remodeling of the PDL as well as understanding the intimate bond of tissue histology and function. Hence, this review outlines the most recent achievements in the field—with a particular emphasis on the biomimetic approach, foreseeing the use of PDLSCs in combination with biomimetic scaffolds and subjected in vitro to controlled native-like mechanical loadings—showing that periodontal tissue engineering represents a promising strategy for innovative therapies in the near future.

## 2. The Role of PDL Cells

**Figure 2:**
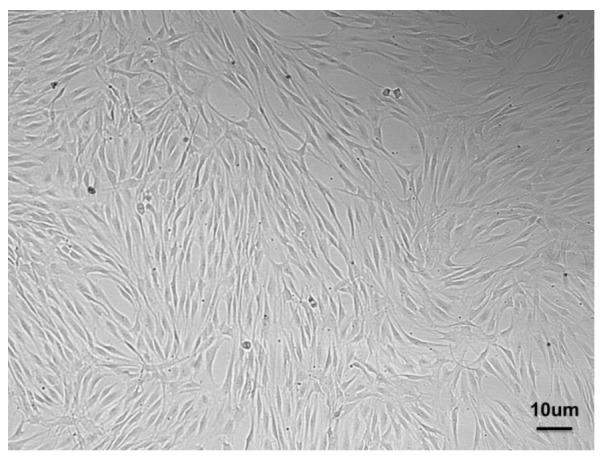
PDLSCs isolated from PDL and expanded in vitro showing the typical spindle shape.

In 2004, Kawaguchi et al. proposed the autograft of bone-marrow-derived mesenchymal stem cells (BMMSCs) to enhance the healing of periodontal defects, which proved successful in a dog model [17]. This approach highlighted the potential of cellular therapy and paved the way to other pre-clinical studies dealing with BMMSCs [18], adipose derived stem cells (ASCs) [19], and PDLSCs [20]. Among all the possible sources of MSCs, PDLSCs (Figure 2)—which are characterized by the expression of many markers resumed in Table 1—may be selected for PDL regeneration owing to their commitment capacity, as they express scleraxis, i.e., a tendon/ligament-specific transcription factor, more than BMMSCs or dental pulp stem cells (DPSCs) and have the potential to form cementum and PDL-like structures [21,22]. Indeed, the preservation of the PDL is essential to achieving proper regeneration of the periodontium and avoiding the ankylosis of the tooth, i.e., direct contact between the root and the alveolar bone.

From this perspective, the role of the transforming growth factor–β1 (TGF-β1) signaling becomes interesting since its activation enables the commitment of cementocytes, while its inhibition promotes fibroblastic differentiation of the ligament progenitors [23]. PDLSCs transplanted into a periodontal lesion in a rat model generated typical PDL-like structures in vivo by forming Sharpey’s-fiber-like collagen bundles that are connected to cementum-like structures [20]. New insights into the molecular regulation of periodontal attachment have been brought by Bai S et al., who investigated the regulatory mechanism of copine 7 (CPNE7) and cementum attachment protein (CAP) in coordination with cytoskeleton arrangement [24]. Regenerating cementum is regarded as key to promoting new fibrous tissue attachment, preserving the PDL, and avoiding tooth ankylosis [25].

Important issues should be considered in order to implement successful PDLSC-based protocols for PDL regeneration. Owing to the reduced volume of the source tissue, the yield of PDLSCs isolated from a single tooth may be poor, and the in vitro expansion of these cells has been linked to morphological changes and diminished expression of genes associated with pluripotency of embryonic stem cells, such as NANOG and OCT4 [26]. Additionally, reducing the ex vivo manipulation of cells is a goal in every cell transplantation procedure since ex vivo expansion of PDLSCs, as well as of ASCs, causes senescence, decline in multipotency, and is subjected to complex regulatory issues under the compelling requirements of good manufacturing practices (GMP) guidelines [27]. Therefore, it becomes paramount to select the best harvest conditions and methods to maximize the number of cells available. The outgrowth method has been suggested to outperform enzymatic digestion in terms of efficiency and preservation of the commitment capacity regarding both the formation of mineralized nodules and the expression of cementoblast-like genes [28]. Consistently, the paper by Abuarqoub et al. compared the features of PDLSCs expanded using either fetal bovine serum (FBS) or platelet lysate (PL) and concluded that the latter outperformed the former [29].

Regarding the possible sources of heterogeneity-influencing PDLSC behavior pertaining to differentiative potential, donor age plays an important role [30,31]. By analyzing donor age impact on PDLSC function, it was observed that PDLSCs from elderly populations exhibited decreased expression of osteogenesis-related genes, such as osteocalcin (OCN), COLL-1, and runt-related transcription factor 2 (RUNX2) as well as reduced osteogenic activity [30]. This has prompted the possibility of banking cells as soon as teeth are extracted to create a reservoir for any future usage. Little is known, however, about the differences between PDLSCs deriving from deciduous and permanent teeth. The latter seem to be less adipogenic than deciduous teeth according to some authors [32,33], while others disagree [34]. Moreover, PDLSCs from deciduous teeth express higher levels of cytokines that regulate the host immunity and secreting factors involved in tissue degradation and catalytic activities than PDLSCs from permanent teeth [35].

Distinguished researchers [36,37] explored the feasibility of using PDLSCs to form cementum and PDL-like structures in proper animal models, reporting very encouraging results. Unfortunately, the enthusiastic outcome of these in vivo studies does not seem to be fully supported by clinical evidence, which is surprisingly scarce. In fact, only one randomized controlled trial [38] assessed the safety and feasibility of using autologous PDLSCs in combination with bovine-derived bone mineral materials for treating periodontal bone defects, but it could not demonstrate any statistically significant difference “between the Cell group and the Control group (*p* > 0.05)”, implying that more studies with incremented numbers will possibly shed light on the matter. It appears that in humans, cells—however important—may alone not be sufficient to regenerate the PDL, which is endowed with a peculiar and rather complex architecture.

**Table 1:** Main markers expressed by PDLSCs.

	MARKERS	REFERENCE
**MSC**	CD105, CD73, CD90, CD44	[39,40]
**STEMNESS**	OCT3/4, NANOG, SOX2	[41,42,43,44]
**PERICYTE**	STRO-1, CD146	[45]
**NEUROECTODERMIC**	Nestin, SOX10, SLUG CD271/p75NTR	[46,47]

## 3. Biomimetic Scaffolds to Reproduce the Micro-Environment of PDL

Traditionally, periodontal regeneration achieved through guided tissue regeneration (GTR) has been based on the concept of avoiding epithelial invasion of the bone defect to be treated by means of barrier membranes to allow PDL and bone repopulation of the dental root. Thus, a specific avenue of research has been paved toward the improvement of these membranes from the original non-resorbable expanded polytetrafluoroethylene (e-PTFE) [48] to the high technology level of recent developments, such as that proposed by Nasajpour et al. [49]. In parallel, the promising potential unleashed by tissue engineering, which relies upon combining biomaterials functioning as scaffolds and stem cells, has opened a range of new therapeutic strategies in the periodontal field. In a pioneering proof of concept, Sonoyama et al. proposed PDLSCs, the resident stem cells of PDL, in association with hydroxyapatite (HA) and tricalciumphosphate (TCP) for forming cementum and PDL-like structures [36]. More recently, Shi et al., after culturing PDLSCs in osteogenic conditions and seeding them on a biphasic calcium phosphate scaffold, observed a periodontal regeneration in the recipient animal constituted by new bone formation and PDL-organized fibers correctly inserted into adjacent cementum and bone, along with neo-vascularization, after 12 weeks [37]. Given these premises, it has become increasingly evident that the PDL is itself the key to attaining the complete regeneration of the periodontium, since this thin tissue of less than 500 μm interconnecting dental root and alveolar bone [21,22] through a series of collagen fiber bundles, is obliterated in PD. Many research efforts have been and are currently spent on identifying the best methods for fabricating biomimetic 3D scaffolds able to reproduce the PDL microenvironment. In Table 2, we report some relevant in vivo studies.

### 3.1. Cell Sheet Technology

One of the first tissue engineering approaches the cell sheet technology, based on culturing the cells in hyper-confluency until they produce their own extracellular matrix (ECM) by forming a cell sheet [58]. Proposed by Okano et al., this technique entails the use of poly-N-isopropyl acrylamide (PIPA Am) as a convenient cell substrate capable of both supporting the growth of a cell monolayer at 37 °C and releasing it below 20 °C without any enzymatic degradation [59]. The adhesion of the cell sheet to the root surface was enhanced through the preservation of the integrin–fibronectin complex [60]. In 2009, Iwata et al. isolated canine PDLSCs and seeded them on temperature-responsive culture dishes until sheet formation. Three-layered PDL cell sheets supported with woven poly glycolic acid (PGA) were transplanted to the exposed dental root surfaces, and bone defects were filled with porous β-TCP, inducing both the regeneration of new bone and the connection of cementum with well-oriented collagen fibers [50]. In 2012, Vaquette et al. combined fused deposition modeling with electrospinning, obtaining a biphasic scaffold with compartments through bone and PDL sheets, demonstrating that the presence of cell sheets promoted periodontal fiber attachment and cementum-like cells [51]. Takahashi et al. demonstrated that PIPA Am is useful for fabricating a brush surface with selective patterns capable of supporting cell growth while preserving orientation [61]. To overcome the pitfalls of single cell sheets in large-scale tissue injuries, Raju et al. proposed 3D complex cell sheets composed of multiple types of cells, attaining the functional connection of collagen fibers to the tooth root and alveolar bone [62]. Similarly, the co-culture of PDLSCs and human umbilical vein endothelial cells (HUVECs) allowed the generation of 3D cell sheet constructs that were wrapped around human tooth roots and implanted into the subcutaneous layer of mice. The presence of HUVECs contributed to the regulation of the thickness of PDL, which was thicker than in mice treated with PDLSCs alone [52]. An unsolved issue of PDL cell sheet technology [63], however, is achieving the directional control of the fibrous network within the constructs. To verify this crucial role of the ECM, as a proof of concept, microfibrous scaffolds were prepared by removing the cellular component from tooth slices using sodium dodecyl sulfate and Triton X-100 and supporting the repopulation and differentiation of PDL cells [64].

### 3.2. The 3D Printing

Among the most promising techniques implemented to physically control the orientations of PDL, researchers have focused recently on additive manufacturing, a technique that allows one to precisely control the macro- and micro-structure of the scaffolds [65,66]. Ideally, this technique can build complex tissues by depositing different materials layer by layer following 3D digital models and can even embed cells directly within the constructs during the fabrication in a process called bio-printing [67]. Hence, the main advantage over traditional tissue engineering protocols relies in the possibility of fine-tuning the creation of tissues to be akin to that of the native cellular micro-environment [68,69]. This approach may be used as a sophisticated mean to reproduce proper fiber orientation, creating specific micro-grooved surfaces for aligning human PDL cells with high predictability [54]. Such is the case of polymeric 3D scaffolds capable of replicating the peculiar micro-patterned histological architecture [70]. In vitro, this arrangement could be maintained for prolonged periods of time in the presence of growing cell populations [25].

#### 3.2.1. Synthetic Polymers and Surface Modifications of Printed Scaffolds

Among the materials suitable for 3D printing, polycaprolactone (PCL) is widely used due to its convenient rheological, mechanical, and biological features [71]. PCL scaffolds endowed with meso/microscale architectural features were also fabricated to form de novo bone–ligament–cementum complexes in vivo [53]. A 3D-printed bone region with grooved pillars seeded with fibroblasts overexpressing bone morphogenetic protein (BMP)-7 was covered with a tooth dentin segment, and was subsequently positioned subcutaneously in a murine model, with a very encouraging outcome [53].

**Figure 3:**
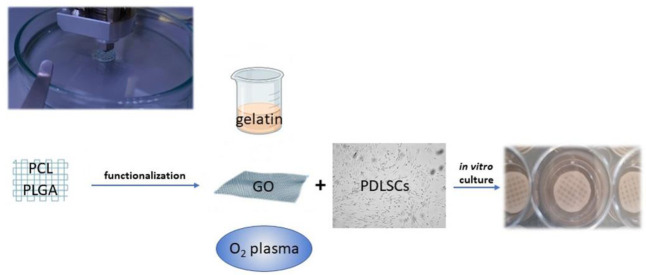
Polymers, such as PCL and PGLA, can be used to print scaffolds functionalized to promote PDLSC adhesion and proliferation. Different strategies of functionalization can be adopted: gelatin nanocellulose can be mixed with PCL or used as an envelope to incorporate other nanoparticles; graphene oxyde (GO) coating increases the hydrophilicity of the PCL surface; oxygen plasma variates the surface roughness.

To improve cell adhesion efficiency on PCL, various surface modification treatments aimed at reducing its hydrophobic interface have been proposed, such as graphene oxide (GO), oxygen plasma, and gelatin coatings (Figure 3) [72,73]. Through plasma treatment, it is possible to variate the surface roughness of nanosized PCL scaffolds, conveniently modulating cell adhesion [74]. Moreover, through electrospinning technique, PCL allows the preparation of nanofibrils or nanocellulose membranes, which can be utilized to encapsulate and carry drugs. For instance, membranes made of PCL encapsulated in gelatin nanocellulose were prepared, and magnesium oxide nanoparticles were incorporated inside them. This system showed high biocompatibility and hydrophilicity that promoted PDLSCs proliferation rates [73].

Vera-Sánchez et al. studied the biocompatibility and potential of a composite coating with GO to induce differentiation of human PDLSCs [75], showing that the GO coating technology increases the hydrophilicity of the PCL surface, promoting cell adhesion. Additionally, poly(d,l-lactide-co-glycolide)/hyaluronic acid PLGA/HA biodegradable microcarriers were treated with GO, improving osteogenic differentiation of stem cells [76]. PCL is fundamental in allowing the printability of the scaffold since it can be conveniently integrated with proper hydrogels functioning as cell carriers and possibly other components, such as a mineralized compartment in a multilayered construct. From an anecdotal point of view, a human case of a large periodontal bone defect treated with a 3D-printed PCL-based scaffold and enriched with platelet-derived growth factors has been reported [77]. Unfortunately, the scaffold was removed after 13 months due to exposure and bacterial contamination. This unsuccessful outcome likely depended on the slow resorbability of PCL and the geometry of the construct, which was too bulky and scarcely interconnected.

Melt electrowriting (MEW), a novel technology particularly suitable for PCL [78], is expected to overcome the common limitations of 3D-printed scaffolds, such as porosity not being well matched to tissue, poor resolution, and inflexible shapes [79]. MEW enables the fabrication of micron- to nanodiameter filaments arranged in highly ordered architectures [80] within multicompartmental scaffolds [81] that may mimic the biochemical composition and/or structural organization of the hierarchical structure of the periodontium, incorporating not only PDL but also its interfacial tissues [82]. By presenting selectively regulatory cues within each compartment, multicompartmental scaffolds can guide cells to form the tissue types desired within the anatomical locations designed [83], promoting cell/tissue in-growth [84]. In 2022, the research group led by William V. Giannobile proposed the design of tricompartmental scaffolds obtained via MEW. Thereby, human PDLFs and primary osteoblasts were co-cultured, achieving “a mineral gradient from calcified to uncalcified regions with PDL-like insertions within the transition region” [85]. As the authors claim, their “process effectively recapitulates the key feature of interfacial tissues in periodontium”, offering “a fundament for engineering periodontal tissue constructs with characteristic 3D microenvironments similar to native tissues”.

#### 3.2.2. Natural Polymers

A viable and natural alternative to PCL is collagen, the primary extracellular PDL protein, which has been employed widely as grafting material owing to its outstanding biocompatibility [86,87]. Unfortunately, collagen alone is not easily printable because of its low viscosity and denaturation temperature [88]. To address this problem, a novel technology called freeform reversible embedding of suspended hydrogels (FRESH) was introduced whereby collagen was deposited in a hydrogel that functioned as a transient mold to be removed non-destructively afterwards [89]. FRESH allowed the printing of collagen parts of human hearts with a satisfactory resolution (20–200 μm) [90].

A good candidate combining antibacterial properties and printability is chitosan, a natural biodegradable polysaccharide already used for guided tissue regeneration [91,92]. A nanohydroxyapatite–chitosan scaffold combined with PDLSCs resulted in effective promotion of bone regeneration in a calvaria bone repair model [93]. In 2018, Varoni et al. [94] prepared a tri-layer scaffold characterized by highly oriented channels aimed at guiding PDL fiber growth through electrochemical deposition. The other compartments were produced with medium- and low-molecular-weight chitosan for regenerating gingiva and bone, respectively. Excellent results supported the feasibility of this resorbable tri-layered structure both in vitro, with high cell survival rates, and in vivo, achieving selective differentiation in terms of mineralized deposits. From this perspective, the development of a new chitosan-based bioink incorporating cellulose nanocrystals, although it has only been tested on murine pre-osteoblasts, is to be regarded as most interesting [95] since it could extend the application of 3D bioprinting for periodontal regeneration to a natural polymer.

#### 3.2.3. Hydrogels

The above-described natural polymers, collagen and chitosan, along with fibrin can also be used as hydrogels, being biodegradable and biocompatible and resembling the original ECM components. The ideal carrier is meant to mimic the ECM, which forms an intricate fibrillar architecture, and can also deeply affect PDLSC colonizing capabilities. Indeed, when seeded on a fibrin sponge, PDLSCs produced abundant ECM, that was positively stained by Alizarin Red S [96]. The effect of a biomimetic electrospun fish–collagen/bioactive–glass/chitosan composite nanofiber membrane (Col/BG/CS) on periodontal regeneration was investigated, showing that the composite membrane promoted cell growth and osteogenic gene expression in vitro, but it was also effective in promoting PDL and bone formation in a canine model [55].

The combination of collagen and methacrylate has garnered growing interest owing to the suitability of the latter for 3D printing; indeed, by adding methacrylate, the collagen may crosslink via UV light in a more controlled way in lieu of using thermal crosslinking. A customized 3D cell-laden hydrogel array with a gradient of gelatin methacrylate (GelMA) and poly(ethylene glycol) (PEG) dimethacrylate compositions showed that the higher the ratio of PEG was, the better the performance of the PDLSCs in cell proliferation and cell spreading on the scaffold [97]. Nonetheless, as inert ECM-based scaffolds alone may be poorly efficient for generating durable tissue repair, they are usually functionalized to release active compounds. PDLSCs sheets combined with platelet-rich plasma were useful for increasing the production of ECM and enhancing cell differentiation [98]. PDLSCs engineered to overexpress platelet-derived growth factor-BB showed increased osteogenic power and were tested in a rat model to induce alveolar bone regeneration [99,100]. The presence of signaling molecules, such as connective tissue growth factor (CTGF), BMP-2, and BMP-7 promote tissue regeneration and cementogenic differentiation [101,102,103]. These three factors have been incorporated into 3D-printed PLGA microspheres, and the results indicated that BMP-7 triggered thicker cementum-like layers, better integration with the dentin surface, and higher expression of cementum protein-1 [56]. In situ tissue engineering (iTE) allowed the production of a iTE-scaffold made with a PLGA/poly (L-lactic acid (PLLA) shell/core structure and functionalized to allow a sequential delivery of b fibroblast growth factor (bFGF), which promotes regeneration of the periodontium [104] and BMP-2, significantly facilitating stem cell homing, proliferation, and periodontal bone regeneration [57,105]. This iTE-scaffold, implanted in a rat model of periodontal defect, demonstrated an anti-inflammatory response, provided adequate blood supply, and achieved the desired bone repair [106]. Regrettably, over the years, safety concerns have hindered the usage of bioactive molecules such as BMP-2 at the high concentrations required to be effective [107], somehow questioning the classic paradigm of tissue engineering based on cells/scaffolds/signaling cues and favoring the development of smart materials [108].

To avoid the functionalization through bio-active molecules, increasing interest in the synthesis of conductive polymers that are also printable—such as poly(3,4-ethylenedioxythiophene): polystyrene sulfonate (PEDOT:PSS) [109], which can be conveniently enriched by poly(vinyl alcohol) (PVA) forming a hydrogel strain sensor [110]—has been conveyed. Conductive hydrogels may become ideal interfaces with the human body, but rarely simultaneously possess the satisfying electrical, mechanical, and adhesive properties shown by the Ti3C2Tx-polyacrylic acid hydrogel that can be printed into complex geometries with high resolution [111].

## 4. Mimicking the Physical Micro-Environment of PDL

During normal oral functions, intermittent occlusal contacts accompanied by pressure from the tongue occur, and the PDL periodically undergoes different combinations of mechanical loading (i.e., compression, stretch, fluid-induced shear stress) that contribute to maintain the PDL homeostasis [112]. The mechanical response of PDL to experienced loading is determined by the combination of the oriented collagen fiber bundles and the distribution of the interstitial fluid, which makes the PDL acting as a shock absorber, increasing the tooth’s ability to withstand loading via the hydrostatic effect [113,114]. This is accompanied by a mechano-biological response of the PDL that, depending on the location under the tooth, changes structure and functions and induces remodeling in the surrounding tissues [5]. Cells detect and transduce the mechanical signals from their membrane to the nucleus through a molecular process named mechanotransduction [115]. Among the most relevant cell membrane mechanosensors, integrins play a fundamental role, mediating direct contact with the ECM. As transmembrane constituents of the focal adhesions (FA), integrins interact with scaffolding, docking, and signaling proteins linked to the actin cytoskeleton [116]. The variable composition of the FA core depends on ECM and mechanical stimuli. Cells modulate their own cytoskeletal architecture in response to applied forces [117] and remain in a sort of tensional homeostasis, i.e., a basal equilibrium stress state [118]. From a molecular point of view, the role of two actin-binding proteins associating with the cytoplasmic tail of the β1 integrin—talin and filamin A (FLNa) [119]—is noteworthy. By interacting with integrins, talin enhances cell adhesion to the ECM [120]. Without mechanical stimuli, talin is fully structured, but while under increasing force regimes, talin exposes progressively more vinculin binding sites (VBS), thus activating more vinculin proteins [121]. Vinculin may mediate reorganization of cell polarity, helping the cell to adapt to increased tensile forces [122]. FLNa competes with talin for binding to β1 integrin [123], and it is thought to antagonize integrin-mediated cell adhesion [124]. For example, Shifrin et al. showed that through the Rac/Pak/p38 signaling pathway, FLNa may prevent apoptosis in PDL in response to tensile forces [125].

**Figure 4:**
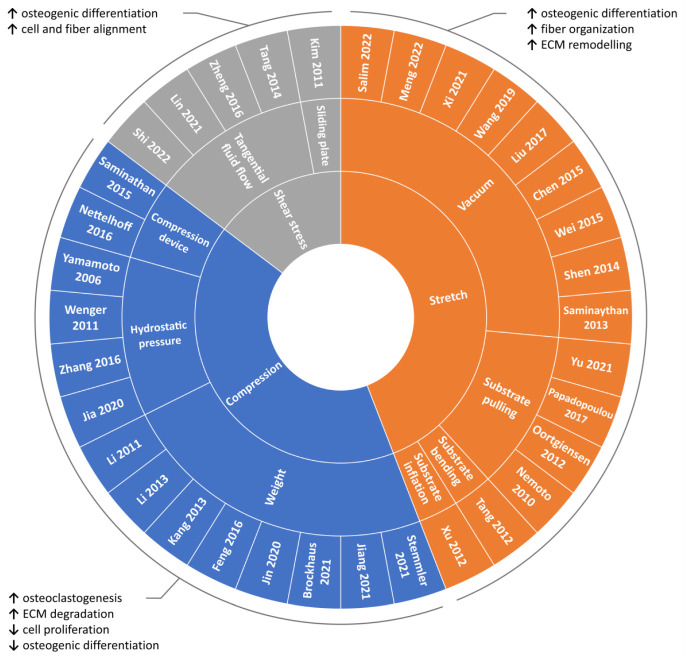
Overview of the reviewed in vitro PDL mechanical loading studies. Schematic summary of the reviewed studies investigating the influence of mechanical loading on PDL cells based on the type of applied stimulation along with main outcomes.

**Figure 5:**
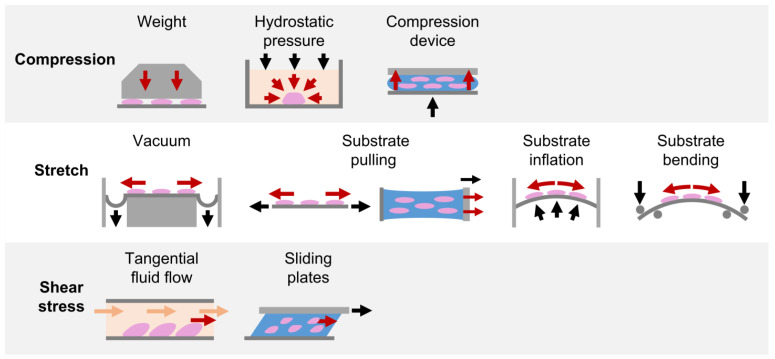
In vitro mechanical loading methods applied for PDL investigations. Schematic representation of the in vitro mechanical loading methods adopted in the reviewed studies for exposing PDL cells cultured in 2D layers or in 3D constructs to compression, stretch, and shear stress stimuli.

Due to the fundamental role played by mechanical loading in vivo, a compelling strategy for directing cell commitment in periodontal tissue engineering may be represented by the reproduction in vitro of the dynamic environment in which PDL cells operate. Several groups investigated the sensitivity of PDL cells (i.e., cells harvested form the PDL, including not only PDLSCs but also more committed cells and even fibroblasts) to mechanical loading and their involvement in periodontal and bone remodeling in vitro. In the following paragraphs, investigations performed in the last decade on in vitro mechanically stimulated PDL cells and constructs are reported per type of force (i.e., compression, stretch, shear stress; Figure 4 and Table S1) and depending on the adopted in vitro mechanical loading method (Figure 5), highlighting the use of conventional two-dimensional (2D) or more physiological 3D cell culture techniques.

### 4.1. Compression

#### 4.1.1. Weight Method

**Figure 6:**
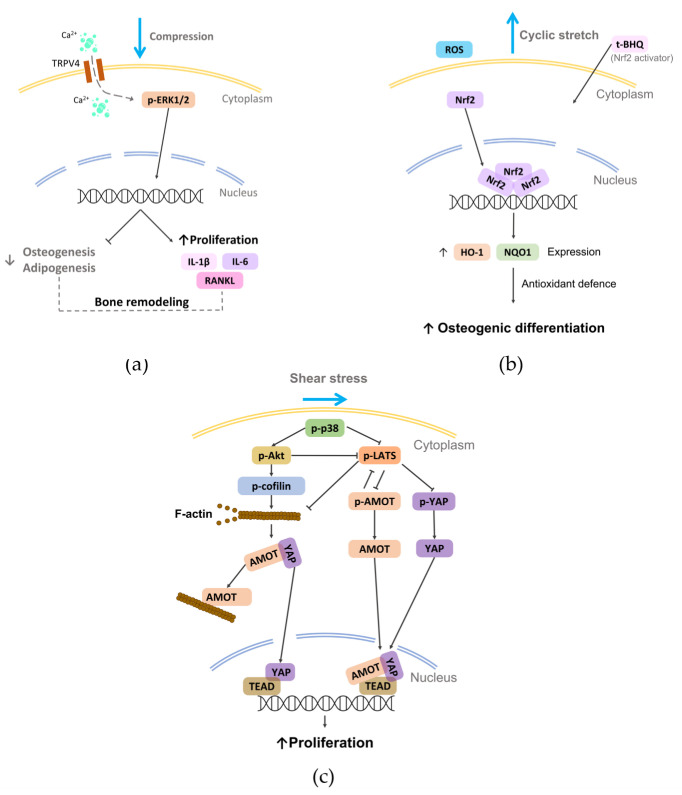
Schematic representation of some mechanotransduction pathways activated on PDL cells by the reported physical stimuli (i.e., compression, stretch, and shear stress). (**a**) External compression applied on rat primary PDLSCs can induce the activation of TRPV4 and consequently modulate bone remodeling [130]; (**b**) external cyclic stretch can promote the osteogenic differentiation of hPDLSCs by activating the Nrf2 [146]; (**c**) shear stress applied to hPDL cells can activate p38, which regulates the nuclear translocation YAP, promoting cell proliferation [162].

This method, based on the use of cover glasses or cylinders containing metal granules, which allows the application of tunable static compressive forces to the culture, was widely adopted to investigate in vitro how continuous compression can influence PDL cells. In 2011, Li et al. established a 3D model of PDL tissue based on hPDL cells seeded on a sheet of porous PLGA scaffold and exposed it to static compression (5–35 g/cm^2^ for 6–72 h), observing a significant induction of osteoclastogenic genes that did not occur when human gingival fibroblasts were used [126]. Moreover, a predominant upregulation of osteoclastogenesis inducers was observed at the early stage (6 h), while osteoclastogenesis inhibitor genes increased at the late stage (24–72 h), although cell proliferation was reduced [127]. In 2013, a comparison between hPDL cells cultured under compressive forces (2.0 g/cm^2^ for 2 or 48 h) in conventional 2D culture dishes or in 3D collagen gel highlighted significant alterations of the expression levels of several genes [128]. In particular, the number of activated integrin–focal adhesion kinase (FAK) was higher in 3D than in 2D culture, supporting that cellular attachment to ECM can strongly influence cellular responses to mechanical forces [128]. In 2016, it was demonstrated that compressive force (1 g/cm^2^ for 24 h) applied to hPDLSCs altered cell morphology and repressed collagen expression, which both recovered after force withdrawal [129]. Recently, continuous compression (0–1.5 g/cm^2^ for 12 h) on PDLSCs could reduce differentiation ability and increase macrophage migration, osteoclast differentiation, and proinflammatory factor expression. Moreover, a universal upregulation of the subfamily V member 4 of the transient receptor potential calcium channel (TRPV4), which regulated osteoclast differentiation by affecting the system receptor activator of nuclear factor kappa-B ligand (RANKL)/osteoprotegerin (OPG) via extracellular signal-regulated kinase (ERK) signaling (Figure 6a) [130], was shown. Brockhaus et al. reported that hPDLFs cultured under compression (2 g/cm^2^ for 24, 48, and 72 h) changed their morphology towards more unstructured, unsorted actin filaments, with a significant reduction of proliferation followed by recovery after 48 h, demonstrating that hPDLFs restore homeostasis and adapt to the compressive force through a lower cell division rate and a slowed cell cycle [131]. Moreover, Stemmler et al. reported that the inflammatory response of hPDLFs caused by periodontal pathogens combined with compressive load (2 g/cm^2^ for 6 h) was supported by the growth differentiation factor 15 (GDF15), which modulated the inflammatory response of PDLFs also regulating the levels of the key inflammatory molecule TNFα [132]. Recently, Jiang and colleagues showed a novel cellular mechanism. Indeed, continuous compressive force (0.5–2.5 g/cm^2^, 12 h) activated autophagy in hPDLSCs that induced M1 macrophage polarization via the inhibition of the AKT signaling pathway, contributing to the force-induced bone remodeling and tooth movement [133].

#### 4.1.2. Hydrostatic Pressure Method

The hydrostatic pressure method exploits air pressure applied on the culture medium for imposing static or fluctuating compressive forces. Exerting a static compressive stress on PDLSCs (100 kPa, for 1, 6 or 12 h) [134] and on hPDL cells (1 MPa or 6 MPa for 10 or 60 min) [135], the expression of genes regulating osteoclastogenesis and osteoblastogenesis was induced. For mimicking the physiological loading during mastication, cyclic hydrostatic pressure was applied (1 MPa, 0.1 Hz, 3 h/day for 2 days) on hPDLFs, showing that the expression of several integrins, collagens, and metalloproteinases was significantly upregulated [136]. Recently, the inflammatory, osteogenic, and pro-osteoclastic effects of different cyclic compressive loading conditions (50–150 kPa, 0.1 Hz, for 1 h/day for 5 days) were investigated by stimulating hPDL cells in an inflammatory environment using a customized bioreactor [137]. According to the level of cyclic pressure, cells released different levels of inflammatory and pro-osteoclastic factors, modulating the downregulation (with150 kPa) or the upregulation (with 90 kPa) of osteogenic genes (alkaline phosphatase (ALP), collagen type I (COLL-1), RUNX2, OCN, osteopontin (OPN), and osterix (OSX)) [138].

#### 4.1.3. Substrate Deformation Methods

Compression can also be achieved through several commercial and customized devices based on substrate deformation, in which an elastic membrane is deformed by force and the cells/constructs cultured on it are exposed to strain. In 2014, a 3D construct composed of hPDL cells seeded into a matrix of hyaluronan, gelatin, and COLL-1 was exposed to cyclic compression (340.6 g/cm^2^ for 1 s every 60 s for 6, 12, and 24 h) by using the commercial Flexercell FX-4000C Strain Unit (Flexcell International Corporation, Hillsborough, NC, USA). Compression increased cell death and the expression of several apoptosis-related genes. ECM genes were mostly upregulated after 6–12 h, but all were downregulated at 24 h, except for the three major ECM-degrading enzymes—MMPs1–3— and the connective tissue growth factor (CTGF), with upregulated matrix metalloproteinase-1 (MMP-1) and tissue inhibitor of metalloproteinases-1 (TIMP-1) protein levels without changes observed in RANKL, OPG, and basic fibroblast growth factor (FGF-2) expression [139]. By using the same device, Nettelhoff et al. exposed hPDLFs to compressive force (5 and 10% for 12 h). The 5% compression induced the highest ALP gene expression and the highest RANKL/OPG ratio, while 10% compression decreased cell viability without promoting apoptosis but resulting in tissue damage [140].

Thus, short-term static compression (almost for 1 h) can promote osteogenic differentiation of PDLSCs, while long-term static compression (for 12 h or longer) can alter the morphology of hPDL cells, may inhibit cell proliferation and the osteogenic differentiation of PDLSCs, and can promote the secretion of osteoclastogenesis-stimulating cytokines and ECM degradation.

### 4.2. Stretch

#### 4.2.1. Substrate Deformation—Vacuum Approach

The vacuum approach stretches the cell-seeded membrane across a loading post by applying vacuum pressure, delivering a tunable biaxial or uniaxial tensile strain. This method is commonly applied by using commercial devices (e.g., Flexercell tension system (Flexcell International Corporation, Hillsborough, NC, USA). In 2013, Saminathan et al. embedded hPDLFs in an 80–100 μm thick 3D collagen membrane and cultured the construct under equibiaxial cyclic stretching (12%, 0.2 Hz, 5 s every 60 s for 6 h/day up to 21 days) to investigate the influence on ECM homeostasis. Mechanical loading did not affect the cell number, but it significantly upregulated the release of MMP-1 and TIMP-1 in the supernatants, suggesting that fibroblasts were remodeling the surrounding ECM [141]. This was confirmed by Chen et al. who exposed hPDLCs to equibiaxial cyclic stretching (12%, 0.1 Hz for 24 h), showing the upregulation of major periodontal ECM genes, such as COL1A1, COL3A1 and COL5A1 [142]. Some studies reported that hPDLSCs cultured in osteoinductive medium under cyclic stretching enhanced the osteogenic differentiation [143,144], both maintaining the same parameters stimulation and reducing the time of stimulation [145] or keeping the same strain magnitude but halving the frequency [146,147]. In particular, Xi et al. demonstrated that cyclic stretching (10%, 0.5 Hz, for up to 36 h) could increase the generation of reactive oxygen species (ROS), which may lead to the osteogenic differentiation of hPDLSCs (Figure 6b) [146]. In 2017, Liu et al., culturing healthy or pathological donor-derived PDLSCs under static strain (6–14% for 12 h), showed that PDLSCs from patients affected by periodontitis were more sensible to physical load than PDLSCs from healthy patients, likely due to the inflammatory milieu [148]. Recently, Salim et al. cultured human PDL cells under static strain (2.5, 5, and 10% for 24 h) and performed in vivo analyses on teeth with and without orthodontic tooth movement (OTM) [149]. Interestingly, they found that chaperone-assisted selective autophagy (CASA) machinery genes (chaperones HSPA8 and HSPB8, the cochaperones BAG3 and STUB1, and the molecule SYNPO2 interacting with BAG3 for autophagosome membrane formation) were inherently expressed in PDL cells and exhibited transcriptional induction upon in vitro mechanical strain and in vivo after OTM. The role of FLNa was also investigated, pointing out that it acts as a flexible actin crosslinker that is stretched under tension and degraded by CASA when damaged, which is consistent with previous works [150,151], further supporting the importance of the dynamic environment as a key factor of the homeostatic maintenance of PDL both in physiologic and treatment conditions [152].

#### 4.2.2. Substrate Deformation—Pulling Approach

The substrate-pulling approach is based on a system that clamps the cell-seeded membrane and imposes uniaxial stretch by a controlled actuator. Adopting the commercial STB-140 STREX cell stretch system (Strex Co., Osaka, Japan), it was reported that a long-term cyclic stimulation (5%, 60 s/returns, resting time = 29 s for 7 days) could increase collagen mRNA and protein expression, suggesting that cyclic stretch on hPDLFs may contribute to the homeostasis of PDL fibers and to the ECM remodeling [153]. In 2012, a 3D construct based on a collagen film laden with rat PDLFs was exposed to uniaxial cyclic stretch (8%, 1 Hz (15 min stretch + 15 min rest) for 8 h/day for 5 days) using a customized device. After mechanical stimulation, the cells were perpendicularly oriented with respect to the stimulation direction and, analyzing several genes’ expression (COLL-1, RUNX2, c-fos, and Cox-2), the authors concluded that PDL cells under loading might tend to have bone-like and, at the same time, tendon-like behavior [154]. Applying the same stimulation for a shorter period (16 h) and in a cyclic or static manner, the cellular orientation could be reached, and three different pathways (ERK, p38 and JNK) were activated [155]. In 2021, Yu et al. demonstrated that exposing a hPDLSCs-laden 3D collagen membrane to uniaxial stretching (20% for 5 days) dramatically enhanced the bioactivity of PDLSC-derived exosomes [156].

#### 4.2.3. Substrate Deformation—Inflation and Bending Approaches

Studies based on the substrate inflation approach were inspired by Howard et al. [157]. A cell-seeded membrane is clamped and deflected by hydrostatic pressure applied to the underside, providing uniform biaxial stretch. In 2012, Xu and colleagues showed that hPDL cells exposed to cyclic stretching (1–20%, 0.1 Hz up to 24 h) appeared aligned perpendicularly to the stretching direction, and the expression of the membrane connexin 43 (Cx43) protein could be modulated in a time- and magnitude-dependent manner via cyclic stretching [158]. By adopting the substrate-bending approach, the osteogenic differentiation of hPDLSCs cultured under tensile stress (3000 μstrain, 0.5 Hz) was reached after 24 h of stimulation, demonstrating upregulation of different osteogenic markers [159].

In summary, the aforementioned studies demonstrated that tensile stress could promote osteogenic differentiation. In particular, cyclic stretch can upregulate the protein and mRNA expression of osteogenic genes and the synthesis of osteoclastogenesis-inhibitory molecules, complemented by an increased expression of the major periodontal ECM genes, leading to the homeostasis and organization of the PDL fibers and to ECM remodeling.

### 4.3. Shear Stress

The effect of shear stress on PDL cells has been poorly investigated in the literature, even though this stimulus is an important cue in the physiological environment. The most adopted method for investigating the effect of shear stress on PDL behavior is based on tangential fluid provided by parallel plate flow chambers. In 2014, Tang and colleagues cultured hPDL cells in osteogenic medium under steady fluid shear stress (12 dyn/cm^2^ for 2 h), showing an early morphologic change and rearrangement of filamentous actin with significant increases in ALP activity and mRNA levels of osteogenic genes and osteoid nodules [160]. Similarly, Zheng et al. exposed hPDL cells to fluid shear stress (6 dyn/cm^2^ up to 12 h), reporting a rearranged cell alignment, an inhibited cell proliferation and migration, and osteogenic differentiation [161]. Very recently, Shi et al. observed that fluid shear stress (6 dyn/cm^2^ for 4 h) promoted cell proliferation by activating mechanotransduction pathways involving the p38 mitogen-activated protein kinases, angiomotin (AMOT), and Yes-associated protein (YAP) (Figure 6c) [162]. Adopting the sliding plate method where the 3D construct is housed between two parallel plates with one of them connected to an actuator that imposes controlled sliding motion and consequent shear stress on the construct, a static shear stress was applied to a construct composed of hPDL cells embedded in a collagen gel. After 24 h of stimulation, cells and collagen fibers aligned in the direction of the principal strain vector [163]. Recently, a model of PDL regeneration based on a fiber-guiding scaffold seeded with PDL cells and subjected to shear stress in a laminar flow-based bioreactor (6 dynes/cm^2^ for 1–4 h) showed increased viability, adhesion, and cytoskeleton arrangement compared to cells in the absence of load [164].

Overall, the above-mentioned findings highlight how shear stress can represent an effective approach for guiding cell and fiber alignment and for promoting osteogenic differentiation.

## 5. Future Perspectives

Exciting advances in engineering and cell biology have begun to address the numerous obstacles hampering the implementation of effective protocols for periodontal regeneration. Despite the flurry of research regarding osteogenic differentiation, however, only a few papers deal with tenogenic differentiation, which is essential to preserve the PDL tissue within the mineralized interfaces of cementum and bone. This lack of knowledge could delay the goal of making available cost-effective, patient-specific treatment options in the future. To this end, it is necessary to assess whether and how mechanical stimulation protocols affect the tenogenic differentiation of PDLSCs, as it is conceivable that various types of physical stimulation, with different application times, are required to achieve the combined modulation of osteogenic and tenogenic activities that allow PDL regeneration. Therefore, in-depth in vitro investigations combined with high-throughput analyses are increasingly necessary to unravel molecular and cellular mechanisms activated by different spatial and physical culture conditions. Finally, from a long-term perspective, developing reliable protocols for periodontal regeneration requires the solution of the following challenges: (1) the definition of the most suitable mechanical stimuli and of the optimal stimulation parameters for promoting tissue regeneration; (2) assessing how the biomechanical load affects the tissues to be regenerated overall; (3) handling the construct at the wound site, avoiding microbial infection/reinfection.

## 6. Conclusions

Despite the remarkable endeavors done in the last years to identify new regenerative treatments for periodontitis, periodontal regeneration remains a tricky challenge. GTR was proven to be as effective as the open flap debridement [165] and seemingly more efficient in regenerating bone than the PDL, which is the key for a true restitutio ad integrum of the periodontium. Cell sheet engineering is technically demanding owing to difficulties in handling and stabilizing the construct, which has greatly limited its clinical application [81]. Several pre-clinical studies [166] have proven the effectiveness of PDLSC-based therapies in regenerating PDL, but the clinical feasibility of this therapeutic approach is far from being achieved. Indeed, well-designed randomized controlled trials (RCTs) are mandatory for assessing the long-term success of innovative procedures, but they must abide by stringent safety and regulatory issues, delaying the implementation of protocols based on PDLSC transplantation in humans. To the authors’ knowledge, only one limited RCT has been retrieved in the scientific literature so far (2022). It reported the clinical safety of the treatment with PDLSCs but with inconclusive evidence regarding PDLSC-based periodontal regeneration [38]. In order to create PDL-cementum complexes, it becomes paramount to tailor the cell environment (mimicking the ECM) by selecting the best scaffold capable of supporting PDLSC growth and differentiation in humans, which implies the adoption of the most up-to-date 3D printing techniques. In parallel, the biomimetic approach demands the use of technological devices for supporting the in vitro maturation of the tissue to be grafted, particularly bioreactors providing controlled physical stimuli to reproduce the native environment [167,168,169]. Indeed, mechanical stimulation can be essential for promoting specific cellular and tissue processes with the final aim of inducing the correct orientation of the fibers forming the PDL and of maintaining tissue homeostasis. In particular (Figure 4): (i) moderate compressive forces promote active tissue remodeling, while long-term static compression can alter the morphology of hPDL cells, inhibiting the proliferation and the osteogenic differentiation of PDLSCs while inducing osteoclastogenesis and ECM degradation; (ii) tensile stress enhances the osteogenic differentiation of PDLSCs, and cyclic stretching can contribute to the homeostasis and organization of the PDL fibers and to the ECM remodeling; (iii) shear stress promotes osteogenic differentiation and guides cell and PDL fiber alignment. Since PDL is a complex multilayer tissue that is physiologically subjected to all these types of forces, a biomimetic in vitro model should both mimic the PDL microstructure and provide, in a controlled manner, the actual combined mechanical loading experienced in vivo. Such an advanced model would allow the mechano-biological behavior of the PDL to be comprehensively unveiled and the best culture protocols for obtaining reliable results in terms of PDL regeneration to be defined. Finally, synergic collaborations between biologists, biotechnologists, biomaterialists, bioengineers, and dentists become urgent for the development of advanced solutions for more reproducible, standardized, and biomimetic studies that could lead in the near future to a successful production of functionally engineered PDL for clinical applications.

## Figures and Tables

**Table 2:** Representative in vivo studies using different scaffolds and PDLSCs to regenerate the periodontal complex.

Type of Scaffold	Cell Type	Production Method	Outcome	Reference
PGA or PCL+ βTCP	OBs, PDLSCs	Cell sheet technology	Periodontal complex	[50,51]
Human tooth root	PDLSCs, HUVEC	Cell sheet technology	PDL fibers	[52]
PCL	PDLSCs	3D printing of fiber-guided scaffolds	Enhancement of the bone volume fraction and of tissue mineral density	[53,54]
collagen/bioactive glass/chitosan membrane	PDLSCs	electrospinning	Periodontal complex	[55]
PCL/PLGA+BMP-2,-7, CTGF	PDLSCs	3D printing	Cementum-like layer formation	[56]
iTE scaffold (core/shell fibrous super-assembled framework+ BMP-2, bFGF)	PDLSCs	iTE	Periodontal complex	[57]

## Supplementary Materials

The following supporting information can be downloaded at: https://www.mdpi.com/article/10.3390/nano12213878/s1, Table S1. List of the reviewed in vitro PDL mechanical loading studies.
