# Supplementary material for: Challenges of Periodontal Tissue Engineering: Increasing Biomimicry through 3D Printing and Controlled Dynamic Environment
Source: Nanomaterials (Basel). 2022 Nov 2;12(21):3878. doi: 10.3390/nano12213878 (PMC9655809; doi:10.3390/nano12213878)
Supplement: Supplementary file 1 [file nanomaterials-12-03878-s001.zip › nanomaterials-1971387-supplementary.pdf]

Table S1. List of the reviewed in vitro PDL mechanical loading studies. The reviewed studies investigating the influence of mechanical loading on PDL cells or constructs are listed reporting the applied mechanical stimulus, the loading method, the cell type, the culture method, the stimulation protocol, and the main results.

Stimulus	Loading method	Cells	Culture method	Stimulation protocol	Results	Study
Compression	Weight	hPDL cells; human gingival fibroblasts	3D culture on PLGA scaffold	5, 15, 25, or 35 g/cm² for 6 h<br>25 g/cm² for 6, 24, or 72 h	Upregulation of mRNA expression of PTHrP, IL-11, IL-8, and FGF-2 (potential osteoclastogenesis inducers) in hPDL cells by force ≥ 25 g/cm²<br>Up-regulation of osteoclastogenic genes in hPDL cells at 6 h<br>Increase of osteoclastogenesis inhibitor genes and reduction of hPDL cell proliferation at 24-72 h	Li et al., 2011 and 2013 [126, 127]
		hPDL cells	2D culture vs 3D collagen gel	2.0 g/cm² for 2 or 48 h	After 2/48 h, changes in the expression levels of 191/280 and 553/519 genes in 2D and 3D cultured cells, respectively<br>Higher number of activated MAPK and FAK pathways in 3D than in 2D culture	Kang et al., 2013 [128]
		hPDLSCs	-	1 g/cm² for 24 h	Alteration of cell morphology<br>Reduction of collagen expression<br>Recovery after force withdrawal	Feng et al., 2016 [129]
		rat primary PDLSCs	-	0-1.5 g/cm² for 12 h	Greater clonogenicity and proliferation<br>Reduced differentiation ability<br>Improved macrophage migration, osteoclast differentiation, and proinflammatory factor expression<br>Upregulation of TRPV4 regulating osteoclast differentiation by affecting the RANKL/OPG system via ERK signalling	Jin et al., 2020 [130]
		hPDLFs	-	2 g/cm² for 24, 48, and 72 h	Change of morphology (disorganization of actin filaments)<br>Inhibition of proliferation and reduction of viability, followed by a recovery and adaptation after 48 h	Brockhaus et al., 2021 [131]

		hPDLFs	-	2 g/cm² for 6 h + periodontal pathogens	Inflammatory response supported by GDF15	Stemmler et al., 2021 [132]
		hPDLSCs	-	0.5-2.5 g/cm² for 12 h	Increase of the autophagy protein LC3, inducing M1 macrophage polarization via the inhibition of the AKT signaling pathway	Jiang et al., 2021 [133]
	Hydrostatic pressure	hPDL cells	-	1 or 6 MPa, 10 or 60 min	Increase of RANKL and OPG mRNA expression	Yamamoto et al., 2006 [135]
		hPDLFs	-	150 psi (~1 MPa), 0.1 Hz, 3 h/day for 2 days	Increase of integrins, collagens, and metalloproteinase expression	Wenger et al., 2011 [136]
		hPDLSCs	-	100 kPa, for 1, 6 or 12 h	Increase of RUNX2 and Sp7 genes expression after 1 and 6 h and of ALP release after 1 h<br>Decrease in RUNX2 and Sp7 genes expression after 12 h<br>No changes in ALP release after 12 h	Zhang et al., 2016 [134]
		hPDL cells	-	50 - 150 kPa, 0.1 Hz, 1 h/day for 5 days	No changes in osteogenic markers expression (at 50 kPa)<br>Increase of osteogenic markers expression (at 90 kPa)<br>Decrease of osteogenic markers expression (at 150 kPa)	Jia et al., 2020 [138]
	Compression device	hPDL cells	3D culture on matrix of hyaluronan, gelatin, and COLL-1	340.6 g/cm², for 1 s every 60 s, for 6, 12, and 24 h	Increase of cell death (12 and 14 h)<br>Increase of ECM genes expression, MMP-1, and TIMP-1 protein levels (24 h)<br>No changes in RANKL, OPG, and FGF-2 expression	Saminathan et al., 2015 [139]

		hPDLFs	-	5 and 10% for 12 h	Decrease of cell viability at 10% of compression<br>Increase of ALP gene expression and RANKL/OPG ratio at 5% of compression	Nettelhoff et al., 2016 [140]
Stretch	Vacuum	hPDLFs	3D culture on 80-100 μm thick collagen film	12%, 0.2 Hz, for 6 h/day, for 24 h, 7, 14 or 21 days	Increase of MMP-1 and TIMP-1 protein levels in the supernatants and of COL3A1, MMP3, TIMP1, and OPG gene expression<br>ECM remodelling	Saminathan et al., 2013 [141]
		hPDLSCs	-	12%, 0.1 Hz for 6, 12 or 24 h	Up-regulation of RUNX2, ALP, and OCN mRNA expression (after 24 h)	Shen et al., 2014 [143]
		hPDLSCs	-	10%, 1 Hz, for 6, 12, 24 or 48 h	Cell alignment in the strain direction<br>Time-dependent increase of miR-21 expression and ALP activity until 48 h	Wei et al., 2015 [144]
		hPDL cells	-	12%, 0.1 Hz, for 24 h	Increase of COL1A1, COL3A1 and COL5A1 gene expression<br>ECM remodelling	Chen et al., 2015 [142]
		Pathological and healthy hPDLSCs	-	6, 8, 10, 12 or 14%, for 12 h	Increase of cell proliferation and osteogenic capacity until 8% for pathological cells and until 12% for healthy cells	Liu et al., 2017 [148]
		hPDLSCs	-	10%, 1 Hz, for 12 h	Cell alignment in the strain direction<br>Up-regulation of RUNX2, OCN and Sp7 expression	Wang et al., 2019 [145]
		hPDLSCs	-	10%, 0.1 Hz, for 24 or 72 h	Increase of ALP activity<br>Upregulation of OPN, RUNX2, COLL-1, ALP, OCN and OSX expression	Meng et al., 2022 [147]

	hPDLSCs	-	10%, 0.5 Hz, for 1, 3, 6, 12, 24 or 36 h	Generation of ROS<br>Nuclear accumulation of Nrf2<br>Upregulation of OPN, RUNX2, COLL-1 and ALP expression and ALP activity after 3h until 36 h	Xi et al., 2021 [146]
	hPDL cells	-	2.5, 5 and 10%, for 24 h	Upregulation of CASA complex component gene expression	Salim et al., 2022 [149]
Substrate pulling	hPDL cells	-	5%, 60 s/returns, resting time = 29 s, for up to 7 days	Decrease of fibrin forming and FACIT collagens mRNA expression (at 48 h)<br>Increase of non-fibril forming collagens (at 48 h)<br>Increase of COLL-1, 4 and 12 from day 2 to day 7	Nemoto et al., 2010 [153]
	rat PDLFs	3D culture on 200 µm thick collagen film	8%, 1 Hz for 8 h/day for 1, 3 or 5 days	Cell alignment perpendicular to the stretching direction (at day 5)<br>Increase of RUNX2 and COLL-1 gene expressions<br>No changes in C-fos and Cox-2 gene expressions	Oortgiensen et al., 2012 [154]
	hPDLFs	-	8%, 1 Hz, for 15, 30, 60, 180 min or 16 h (static vs cyclic strain)	Cell alignment perpendicular to the stretching direction under cyclic strain (at 16 h)<br>Activation of ERK, p38 and JNK signalling pathways by cyclic strain (at 15 min), the activation returned to the basal level at 180 min<br>Increase of C-fos expression after 15 min of cyclic and static strain (higher expression in cyclic)	Papadopoulou et al., 2017 [155]

		hPDLSCs	3D culture on collagen membrane	20%, for 5 days	No changes in cell viability<br>Increase of the bioactivity of the PDLSC-derived exosomes	Yu et al., 2021 [156]
	Substrate inflation	hPDL cells	-	1, 10 and 20%, 0.1 Hz for 30 min, 1 and 24 h	Cell alignment perpendicular to the stretching direction (at 24 h)<br>Increase of Cx43 gene expression (after 24 h at 10 and 20%)	Xu et al., 2012 [158]
	Substrate bending	hPDLSCs	-	3000 µstrain, 0.5 Hz, for 3, 6, 12, or 24 h	Increase of Satb2, RUNX2 and OSX mRNA expression and protein levels	Tang et al., 2012 [158]
Shear stress	Tangential fluid flow	hPDL cells	-	12 dyn/cm² for 2 h	Promotion of osteogenic differentiation<br>Increase of ALP activity, expressed osteogenic genes and mineralized nodule formation	Tang et al., 2014 [160]
		hPDL cells	-	6 dyn/cm² for 2, 4, 8, 12 h	Induction of osteogenic differentiation (>8 h)<br>Cell alignment along flow direction (2-8 h)<br>Inhibition of proliferation and migration (12 h)	Zheng et al., 2016 [161]
		Immortalized hPDL cells	3D culture on microfibrous scaffold of COLL-1	6 dyn/cm² for 1, 4 h	Increase of cell spreading, adhesion, and viability	Lin et al., 2021 [164]
		hPDL cells	-	1, 3, 6, or 9 dyn/cm² up to 4 h	Promotion of cell proliferation (1-6 dyn/cm²)<br>Inhibition of cell proliferation (9 dyn/cm²)<br>Maintenance of cell viability<br>Increase of F-actin filament density	Shi et al., 2022 [162]

					Promotion of actin cap expansion, nuclei compression, and increased nuclear pore size<br>Activation of ERK, p38, and JNK<br>Time dependent regulation of AMOT and YAP localization	
	Sliding plate	hPDL cells	3D culture on COLL-1 gel	Static shear strain resulting in 1.36 ± 0.53 Pa for 24 h	Alignment of collagen fibers and cells along principal strain vector	Kim et al., 2011 [163]

Acronyms: Alkaline phosphatase (ALP); Angiomotin (AMOT); Basic fibroblast growth factor (FGF-2); Bone morphogenetic protein 2 (BMP-2); Chaperone-assisted selective autophagy (CASA); c-Jun N-terminal kinases (JNK); Collagen type I, alpha-1 (COL1A1); Collagen type III, alpha-1 (COL3A1); Collagen type V, alpha-1 (COL5A1); Connexin 43 (Cx43); Cyclooxygenase-2 (Cox-2); Extracellular matrix (ECM); Extracellular signal-regulated kinase (ERK); Fibril-associated with interrupted triple helices (FACIT); Focal adhesion kinase (FAK); Growth differentiation factor 15 (GDF15); human periodontal ligament (hPDL); human periodontal ligament fibroblasts (hPDLFs); Human periodontal ligament stem cells (hPDLSCs); Interleukin 11 (IL-11); Interleukin 8 (IL-8); Matrix metalloproteinase-1 (MMP-1); Matrix metalloproteinase-3 (MMP-3); MicroRNA-21 (miR-21); Microtubule-associated protein 1A/1B-light chain 3 (LC3); Mitogen-activated protein kinase (MAPK); Nuclear factor erythroid-2-related factor-2 (Nrf2); Osteocalcin (OCN); Osteoprotegerin (OPG); Osterix (OSX); p38 mitogen-activated protein kinases (p38); Parathyroid hormone-related protein (PTHrP); Reactive oxygen species (ROS); Receptor activator of nuclear factor kappa-B ligand (RANKL); Runt-related transcription factor 2 (RUNX2); Serine/threonine-specific protein kinases (AKT); Sp7 transcription factor 7 (Sp7); Special AT-rich sequence-binding protein 2 (Satb2); Tissue inhibitor of metalloproteinases-1 (TIMP-1); Transient Receptor Potential Cation Channel Subfamily V Member 4 (TRPV4); Yes-associated protein (YAP).
